# A stepped wedge cluster randomized trial to evaluate the effectiveness of a community leader-driven kit-based diabetes self-management education approach in improving diabetes control and care: study protocol for the DElhi Diabetes INTervention Trial (DEDINTT)

**DOI:** 10.1186/s13063-023-07712-3

**Published:** 2023-10-17

**Authors:** Jitender Nagpal, Swapnil Rawat, Lovely Gupta, Avantika Negi, Divya Shashi Oraon

**Affiliations:** Sitaram Bhartia Institute of Science and Research, B-16 Qutab Institutional Area, New Delhi, 110016 India

**Keywords:** Diabetes, Self-management education module, Sw-CRT, Delhi

## Abstract

**Introduction:**

Diabetes self-management education (DSME) helps patients self-manage their condition and improve outcomes/quality of life. However, access to DSME is limited, particularly in low-income areas. This study aims to develop a DSME training kit (EK-DIN), understand barriers to implementation, and evaluate the effectiveness and sustainability of community leader (CL)-based rollout using a stepped wedge cluster randomized trial format.

**Methods and analysis:**

The mixed methods study will begin with a qualitative study to evaluate the facilitators and barriers towards CL-based DSME. The in-depth interview notes will be transcribed for thematic analysis. These results will be utilized for a stakeholder’s workshop to develop the EK-DIN kit, a patient-interfacing app, and an implementation plan. Rollout will be conducted in 30 clusters in Delhi, preselected by the DEDICOM-II survey in 5 steps (6 clusters every 3 months: 2 each from each socio-economic category; randomly selected per sequence). A CL from each cluster will be trained in using the EK-DIN kit/app over 1 month. The trained CL will conduct DSME sessions among the cluster residents using the EK-DIN kits provided fortnightly for 3 months. Compliance and blood parameters data will be collected at baseline, 3 months after the intervention, and every quarter thereafter till completion. Change in HbA1c before and after the intervention will be evaluated as the primary outcome using the swCRTdesign package for R version 4.0.2 and the swSummary function. The sustainability of the effects will be evaluated using the change in quarterly parameters after intervention completion.

**Discussion:**

A positive result will set the template for a generalizable public health intervention with proven community effectiveness, sustainability, cost-effectiveness, and positive quality-of-life impact. While a negative result will require the testing of alternative approaches, it would still add substantially to existing knowledge on the subject. Given the diverse socio-cultural setting in which the trial is being proposed and the high power of the study, the results (positive or negative) should be widely applicable and have policy implications.

**Trial registration:**

CTRI/2023/07/054963. Date of Registration: 7th July 2023.

## Administrative information

The numbers in curly brackets in this protocol refer to SPIRIT checklist item numbers. The order of the items has been modified to group similar items (see http://www.equator-network.org/reporting-guidelines/spirit-2013-statement-defining-standard-protocol-items-for-clinical-trials/).

**Table:** 

Title {1}	A stepped wedge cluster randomized trial to evaluate the effectiveness of a community leader-driven kit-based diabetes self-management education approach in improving diabetes control and care: protocol for the DElhi Diabetes INTervention Trial (DEDINTT)
Trial registration {2a and 2b}	CTRI/2023/07/054963
Protocol version {3}	DEDINTT Protocol v 2.0 dated 01/03/2023
Funding {4}	The study was funded from Indian Council of Medical Research (ICMR), New Delhi India (5/4/8–6/Obs/JN/2022-NCD-II)
Author details {5a}	Sitaram Bhartia Institute of Science and Research, B-16 Qutub Institutional Area, New Delhi, 110016, India
Name and contact information for the trial sponsor {5b}	Sitaram Bhartia Institute of Science and Research, B-16 Qutub Institutional Area, New Delhi, 110016, India
Role of sponsor {5c}	Role of study sponsor and funders: The trial sponsor is the host institution. The sponsor and funders have no role in study design; collection, management, analysis, and interpretation of data; writing of the report; and the decision to submit the report for publication

## Background

Type 2 diabetes is a major public health problem, with an estimated 537 million adults worldwide. In India alone, the numbers exceed 101 million and are expected to increase to 134 million by 2045 [1]. Despite its documented high and rising prevalence, serious long-term complications, and established evidence-based guidelines for management, the translation of practice recommendations to care is still deficient [1, 2]. This, in turn, contributes to a high risk of complications, cost, and mortality.

With this background, we undertook two community surveys on the Quality of Diabetes Care and associated cardiovascular risks in Delhi in 2005–2006 [3] and, more recently, in 2018–2020 [4] (DEDICOM and DEDICOM-II; funded by ICMR). Both house-to-house 30-cluster surveys showed that the quality of diabetes care delivered to diabetes patients in Delhi was consistently poor. Although not directly comparable due to differing population sets, it is noteworthy that the percentage of subjects with extremely poor diabetes control (HbA1c > 10) was significantly higher in the second survey compared to the first one. This implied a progressively deteriorating situation and emphasized the urgent need for an effective intervention plan to address the problem.

An important lacuna in the existing strategy [5] for diabetes management [6] is that it relies on setting up care services and puts the onus of delivery of quality care on the public health care delivery system. While the availability of good healthcare services is an important determinant of disease management [6], it has been amply demonstrated that diabetes lifestyle modification (LSM) and diabetes self-management education (DSME) are the two most critical determinants of success beyond drugs and tests [6]. Empowering the individual in a patient-centric manner improves awareness and helps with risk modification and care-seeking behavior in a manner complementary to good medical advice. However, attempts to deliver DSME and LSM at scale through the doctor/diabetes educator approach [7, 8] have met with limited success leading to the search for unconventional options. Several systematic reviews [9–11] have documented that group-based education is more effective than individual-based interventions in improving self-efficacy, glycemic control, lipid profile, blood pressure, body weight, and psychosocial outcomes. Several eminent international studies have also established the utility of community-based, peer-led prevention programs globally and showed improvement in glucose and metabolic control [6, 9, 12, 13]. Digital interventions alone have also been found to be effective globally [14]. However, there is wide variability in such results emanating from local contextual factors/skill levels of trainers. Hence, we hypothesize that a combined approach merging the peer-led and digital streams, and standardizing intervention delivery will maximize community effectiveness.

We are therefore proposing the current work to locally co-develop an educational kit for diabetes intervention (EK-DIN; meaning One-Day in Hindi). We will then evaluate its effectiveness in improving the quality of diabetes care when EK-DIN is used for diabetes self-management education by community leaders. The rollout and evaluation will be done as a stepped wedge cluster randomized trial conducted among the re-consenting participants of the DEDICOM-II survey.

## Study objectives

### Primary objective

- To co-develop a culturally embedded and locally tailored novel self-management education module with known diabetes patients (EK-DIN kit and app) and to evaluate its effectiveness when implemented through a community-leader group-based format in improving the quality of diabetes care (biochemical parameters and preventive processes).

### Secondary objectives

- To evaluate the effectiveness of DSME implemented using EK-DIN through a local community leader group teaching format in reducing cardiovascular risk among known diabetes patients.
- To compare the effectiveness of EK-DIN in improving the quality of diabetes care between socio-economic categories.
- To evaluate the cost-effectiveness of the EK-DIN kit when implemented through a community leader format.
- To assess the quality-of-life impact of a culturally embedded and locally developed novel self-management education module (EK-DIN).

## Methods

### Study design and setting

**Fig. 1:**
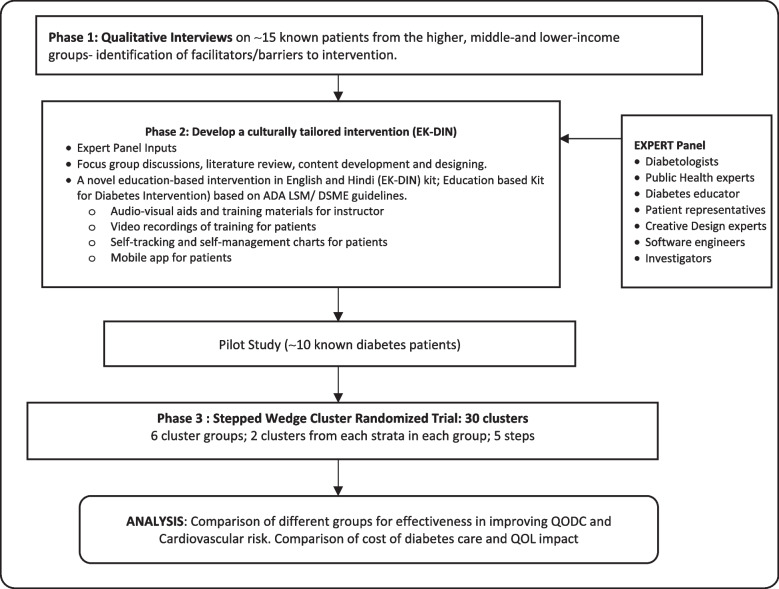
Summary of study design

This study is proposed as a sequential mixed methods design. A summary of the study design is depicted in Fig. 1. As presented, the study comprises three phases—the first phase involves qualitative study to understand barriers and facilitators to intervention adoption among diabetes patients, the second phase involves intervention development by participative stakeholder engagement, and the third phase involves a subsequent intervention evaluation phase involving a stepped wedge cluster randomized trial (Sw-CRT) in Delhi among the participants of the DEDICOM-II survey, India.

### Procedure

#### Phase 1: Qualitative work

As a first step, we propose to conduct qualitative work among ~ 15 diabetes patients from each socio-economic category or till saturation is achieved. This will be used to evaluate the existing knowledge of the subjects about diabetes, their perception of how they could be helped to manage their condition better, and to document the potential facilitators and barriers for successful intervention. Gathering this information would enhance our understanding of the perceived need for the intervention and help us define more patient-centric outcomes. Incorporation of the patient perspective would also help to fine-tune the study design towards improving the sociocultural acceptability and improving compliance with the proposed intervention/follow-up. In this phase, in-depth interviews will be conducted on known diabetes patients with prior informed consent. The audio-recorded consent will be taken by the interviewer before starting the interview. Interviews will be audio-recorded for subsequent transcription. A semi-structured topic guide comprising several open-ended, semi-structured, pre-designed questions will be developed and piloted to direct interviews while remaining sensitive to unsolicited themes. Probes will be used where necessary. An interviewer with prior qualitative research training will conduct the interviews using an “inductive” approach in English or Hindi. Data will be collected in patients’ homes, assuring natural settings and maximizing patient comfort. Thematic analysis will be conducted, and this information will be used to guide phase 2.

#### Phase 2: Development of intervention (EK-DIN kit/app and implementation plan)

Building on the learnings from Step 1, a locally and culturally tailored intervention guided by the ADA guidelines [15] on lifestyle modification (LSM) and diabetes self-management education (DSME) will be developed. The intervention will have three components—an education-based kit for diabetes intervention (EK-DIN), an analogous app, and an implementation plan. A stakeholders panel will be constituted, including diabetologists, public health experts, diabetes educators, patient representatives, creative design experts, software engineers, and investigators. They will conduct focus group discussions towards designing the EK-DIN kit in English and Hindi for use by community leaders for delivering DSME and LSM. EK-DIN will include audio-visual aids and training materials, including the use of glucometers and self-monitoring BP instruments. Software experts will be briefed in the FGDs towards developing an app capable of hosting LSM and DSME audio-visual content, a self-tracking interface for self-review of blood sugars and other reports, and provision for timely reminders on due dates for investigations/consults in a bilingual patient-centric format. An implementation plan, including the preferred types of trainers, locations of the training, days of activities, and the language of training, will also be finalized in the focus group discussions.

#### Phase 3: Implementation of the intervention (stepped wedge cluster randomized trial (Sw-CRT))

The community intervention phase of the study will begin with a pilot on 10 known diabetes subjects from a nearby locality. The information gathered through the pilot will be used to further fine-tune the rollout plan. After the pilot, the rollout will target to recruit 350 diabetes patients from among the subjects of the DEDICOM-II survey [4]. It is expected that it will be possible to recruit ~ 12 subjects per cluster from among the 20–30 subjects/cluster recruited for the earlier survey [4]. All earlier clusters and participants will be eligible for recruitment in the proposed study and will be approached for informed consent.

**Fig. 2:**
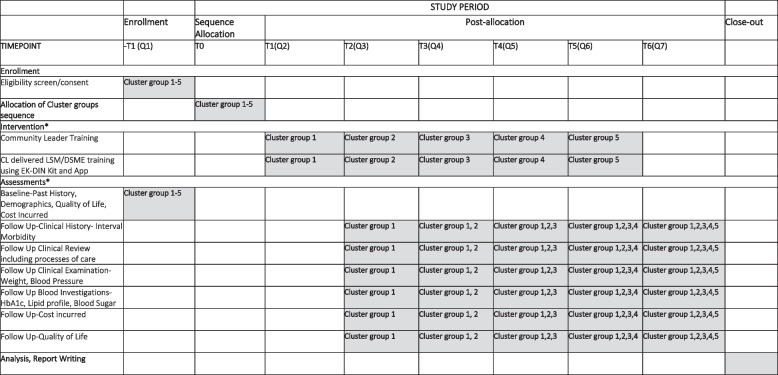
Stepped wedge cluster randomized trial: SPIRIT flow diagram *Intervention and assessments explained in Fig. 3

**Fig. 3:**
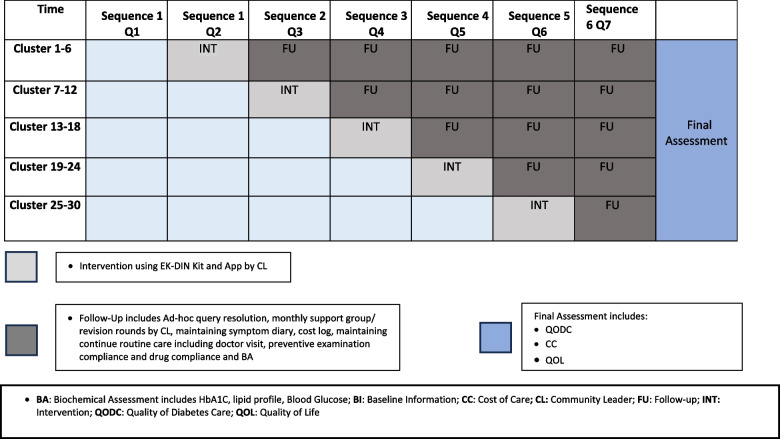
Trial rollout sequence

The intervention will be rolled out using the stepped wedge cluster randomized trial format, as summarized in Figs. 2 and 3. As presented in this design, clusters enter the intervention arm sequentially, contributing control and intervention data, unlike traditional cluster-randomized clinical trials. The design involves 5 groups of clusters (6 clusters in each group; 2 each from lower, middle, and higher strata) with sequential cluster group crossover from control to intervention until all are exposed. Each cluster group will be pre-specified based on geographical contiguity. The order of rollout in the cluster groups will be determined by a computer-generated random sequence. The sequence will be concealed in opaque sealed envelopes till use. While blinding is not considered feasible at the participant/field staff level, the laboratory processing, data entry, statistical analysis, and investigators will be blinded to the sequence of intervention.

As presented in Fig. 3, at Step 0, each cluster group starts as a control condition, with the first cluster group initiating the preparation phase and contributing control data. In Step 1, cluster group 1 enters the intervention period. Three months later, another cluster group (6 clusters) enters the intervention period. This process continues until all clusters enter the intervention. Hence, the stepped-wedge trial design involves 3 months of data in pre-intervention and 18 months in post-intervention conditions for cluster group 1, followed by 6 months in pre-intervention and 15 months in post-intervention conditions in cluster group 2, and so on. All 30 clusters will be completed in 5 steps. The study design has been specifically chosen due to the documented inherent sample size, ethical, logistical, and naturalistic advantages of a wedge cluster rollout [16] and to evaluate the baseline status, impact of intervention, and sustainability of any benefits achieved.

For each cluster group, participants of the DEDICOM-II survey will be invited for recruitment by the research team in accordance with the following selection criteria.

### Eligibility

#### Inclusion criteria

- Subjects recruited in our earlier survey of DEDICOM-II
- Known case of type 2 diabetes mellitus > 1 year (diagnosed by a registered medical practitioner using at least one blood glucose estimation)

#### Exclusion criteria

- Subjects with cancer, renal, hepatic, or intestinal disease requiring continuing treatment or hospital admission (> 1 week in last 1 year)
- Subjects with inability to communicate (mental illness or physical disability)

A written informed consent form will be taken from all the participants prior to the recruitment by the research team. A baseline evaluation of eligible participants will be done, including the HbA1c, lipid profile, blood pressure, and compliance to the frequency of processes of care like eye examination, foot examination, and urine testing. A baseline evaluation of the quality of life using the QOLID tool [17] and cost of care will also be conducted. Quarterly evaluations for clinical assessment, blood investigations, and interval morbidity will continue in all groups. In the month preceding rollout in each cluster, a community leader (CL) will be identified.

### Community leader (CL)

CL will be from among the prominent interested diabetes patients involved in the local administration (RWA president or village head etc.; preference will be given to any person with any medical background like doctor, dietician, or nurse). The CL will be trained (training of trainers) in how to educate for LSM/DSME and improve your own care using EK-DIN over a 1-month period (education sessions of minimum 50 h). The value of the CL in delivering the intervention is expected to stem from their direct experience with the community and participants’ living situations. They will be capable of support and empathy which often is difficult for professionally trained individuals to provide. They will have a first-hand understanding of the myths, beliefs, and cultural remedies that may interfere with the adoption of health recommendations.

As the intervention in the cluster begins, the CL will then conduct 2-h DSME sessions among the particular cluster residents using the EK-DIN kits provided at a pre-specified site fortnightly for 3 months. The classes will be conducted on a public holiday, will be locally advertised, and will be open to all interested. Family members, friends, and/or caretakers will be encouraged to attend. The blood glucose monitoring logs of each participant will be reviewed at the beginning of each session. A mop-up round will be conducted for the subjects who missed the training. In case any participant is unable to attend the 2 rounds of training, video consult modalities will be utilized to administer the intervention. After training, each participant will be provided with self-tracking sheets in manual/electronic format as envisaged and developed in phase 2. Whenever the CL notes that the participants are not meeting the treatment goals, they will encourage the patients to follow up with their primary care providers, but the CL will not be permitted to make any medication/management recommendations.

### Training and quality control

The principal investigator (PI) and the research scientist will conduct study personnel-training sessions on the study protocol and standard operating procedures at the co-ordination center before the initiation of the study including Good Clinical Practice, Human Subject Protection, and Responsible Conduct of Research. The host institute will provide all necessary support for the investigators and in establishing a co-ordination center including seating and training facilities and logistics. The PI will supervise the project implementation by holding biweekly meetings with all the field staff and monthly meetings with all the investigators. The PI will be responsible for upholding the sanctity of the protocol. A Trial Oversight Committee, including subject experts, trial experts, and lay persons, will review the trial’s progress every quarter and provide suggestions for required course corrections. Research fellows will be responsible for identifying potential recruits and obtaining consent. Data will be managed by the research scientist, project co-ordinator, and statistician and saved securely under password-protected access. All clinical data endpoints like interval morbidity will be adjudicated by the Endpoints Adjudication Committee comprising of two diabetologists and a cardiologist independent of the research group and blinded to the intervention/site allocation.

### Duration of treatment and follow-up

After the completion of the intervention, the subjects will be retested for the same parameters (including blood tests) every 3 months till the trial endpoint (18 months for cluster group 1). In the follow-up period, ad hoc query resolution and monthly support group/revision rounds will be conducted by the CL in the cluster till the end of the trial. Each participant will be called by telephone for each session to encourage attendance. All participants will undergo quarterly blood sampling, preventive examination compliance, and drug compliance monitoring every quarter by the research team in coordination with the CL. All participants will be provided with a symptom diary to document the medical issues faced by them through the study period and the direct and indirect costs incurred on diabetes care through the study period. All the participants will be recommended to continue the routine diabetes care, including medicines and doctor visits with their existing care provider and/or preventive checks, and will be assessed for quality of life using the pre-validated QOLID tool at baseline and last follow-up [17]. Cost of care data for both groups will be documented using the cost logs to allow for cost-effectiveness calculations. Any noted new morbidity will be referred to the primary care provider, and the intervention advice will be modified in accordance with the medical advice, even if unblinding is required. Data collection from all participants will proceed as planned irrespective of compliance or shift in residence (by virtual means if required). If consent is withdrawn during follow-up, existing data available with the team till the date of withdrawal will be used as available.

### Biochemical analysis

Blood sample (5 ml) will be drawn by a phlebotomist into three vacutainers (lipid profile, blood glucose, and HbA1c) and transported in ice from the field site to the laboratory within 2 h. The HbA1c sample (EDTA) will be stored at 4 °C until processing (within 24 h). The other vacutainers will be centrifuged at 1310 g. Lipid profile and blood glucose estimation will be done using a Hitachi 902 analyzer. HbA1c will be tested by low-pressure liquid chromatography (Biorad Diastat analyzer; DCCT aligned). Some of the samples (5%) will be randomly re-run to ensure quality control.

### Sample size considerations

The sample size was estimated using the stepped wedge cluster randomized design package (swCRTdesign) in R software (version 4.0.2) [18]. The following information was utilized from our recent DEDICOM-II survey: thirty clusters, a mean HbA1c of 8.9%, standard deviation 4.3, and an intra-cluster correlation of 0.09. Assuming an alpha error of 0.05 and a power of 0.9, it was estimated that a five-step design, using 12 subjects per cluster and with 6 clusters per sequence (2 from each stratum; a total of 360 subjects) will be required to detect a significant mean difference of 1% (~ 10 mmol/mol) in HbA1c level. The study would also be adequately powered (0.8) for comparisons between socio-economic classes with the same consideration using 2 clusters per step per strata.

## Statistical analysis

### Qualitative data

Interview transcripts will be transcribed and translated into English, cross-checked against the original recording, and coded using ATLAS.ti 7.2 or NVIVO 14.0 software for thematic analysis. The research team will code the data into provisional themes in accordance with the six phases of Maguire and Brid [19]. The analysis will be iterative, moving bi-directionally through the phases. The provisional themes will be reviewed for nuances and sub-themes before defining and naming the themes. the defined themes/subthemes will then be mapped onto the factors listed in Andersen’s Behavioural Model (ABM) [20] to understand the barriers and facilitators to the proposed intervention and the Health Beliefs Model to evaluate diabetes-related perceptions of the community. Any additional themes will be recorded as new unmapped items.

### Quantitative data

Change in HbA1c before and after the intervention will be evaluated, accounting for SW-CRT design using the swCRTdesign package for R version 4.0.2 and the swSummary function [18]. The sustainability of the effects will be evaluated using the change in quarterly parameters after the intervention is completed. Differences between socio-economic strata will be compared as sub-group analyses depending on the income category of the clusters. Missing data will not be imputed.

#### Expected outcomes

##### Primary outcome measure

Comparison of the HbA1c levels between the groups 3 months after the start of intervention.

##### Secondary outcomes

These will be compared at the trial endpoint. The trial endpoint will vary from 6 to 18 months after intervention for various cluster groups, as presented in Fig. 3.

- Comparison of HbA1c in the groups at “trial end point”
- Comparison of Lipid profile, blood pressure, drug compliance, and preventive checks between the groups
- Comparison of sustainability of the gains in the groups

##### Economic, social, and qualitative outcome measures

- Cost-effectiveness of the intervention
- Quality-of-life (by QOLID) impact of the intervention

### Data management

Data will be collected under the regulation of the data protection and management guidelines provided by the Governments of India. It will be securely stored in an e-database using Access. Monthly checks and data completeness and outliers will be conducted by the research scientist and data manager.

### Confidentiality

All paper case report forms or source documents will be filed and stored in designated cabinets. All data will be anonymized at entry, stored on a secure password-protected database, and shared only with the research team for the purpose of this research only.

### Dissemination plans

We plan to disseminate results to the funders, patients, and other key stakeholders, including policy makers and experts. This will be done through databases, trial registers, social media, reports, conferences, and publications in peer-reviewed journals. Lay summaries will be prepared to share with participants and policy makers. Stakeholder workshops including patients’ representatives will also be conducted for discussion and dissemination purposes.

## Discussion

The proposed study provides a unique opportunity of working with a pre-engaged community to co-develop a culturally and locally tailored self-management module-based kit for diabetes intervention (EK-DIN) and related app and to subsequently test its effectiveness in improving diabetes control (glycemic, lipid, and blood pressure) and processes of care. A positive result from the study could modify our approach to improving diabetes care from the traditional doctor practice-based route to empowering the patient to be the driver of change. The intervention is intended to be scalable and cost-effective as it sidesteps the resource requirements of a doctor/diabetes educator approach and is likely to be effective even in areas where expert health care is unavailable. The intervention by using a kit and app also avoids the wide variations seen in traditional CL approaches and provides a degree of standardization to the educational intervention.

The study has several unique and important strengths. The trial is preceded by comprehensive qualitative work to evaluate barriers and facilitators to the intervention and seeks to co-develop/fine-tune the intervention kit/app and plan with stakeholders. The trial phase uses an epidemiologically robust stepped wedge cluster randomized methodology with all the advantages of a community trial while minimizing sample size and resource requirements. The trial tests a community-leader-based approach with a standard training kit for effectiveness in resolving a traditionally difficult problem in a resource-constrained setting like India. The trial is proposed in Delhi, which has a large population of diabetes patients of all socio-economic strata, enhancing the generalizability of results. The study follows through in the same population and clusters enrolled in a previous survey providing robust pre-trial estimates and improving the chances of engagement with a pre-primed population. However, there are some limitations. The study is limited to Delhi and is proposed in a pre-sensitized population already covered in a previous survey. While this enhances the feasibility of the study, it limits the generalizability of the findings in dissimilar settings like elite or rural or tribal regions. In elite settings, the acceptability/identification of community leaders might be a problem. In tribal/rural areas, language/cultural barriers may differ and training/engagement of community leaders might be a challenge. While the study deliberately seeks to side-step the existing norms of DSME directly through doctors and educators this may create resentment due to conflict of interest in some areas confounding the results.

Performing a survey with the proposed design could potentially involve several practical and operational issues. First, the logistics of implementing the intervention in different far-off communities at different times can sometimes be challenging. This includes ensuring that all necessary resources and personnel are available at the right times. As specified in the methodology, we intend to mitigate this by pregrouping geographically proximate clusters from each stratum minimizing logistic efforts. Secondly, maintaining consistency in the intervention and data collection procedures across different communities and time points is crucial for the validity of the study. Quality in data collection will be ensured by rigorous training and monitoring of field staff, setting up of End-points Adjudication Commmittee and a Trial Oversight Committee. Thirdly, unforeseen events or changes in community behavior could affect the implementation of the intervention or the collection of data. For this, we intend to re-energize dormant engagements by involving local leaderships and revisiting previous participants prior to rollout.

The results of the proposed study will be interpreted in the context of existing literature related to the effectiveness of group-based diabetes education interventions, peer-led interventions, and digital health interventions. Several systematic reviews have documented that group-based diabetes education is more effective than individual-based interventions. In 2005, a Cochrane systematic review assessed the effects of group-based training on clinical, lifestyle, and psychosocial outcomes in people with type 2 diabetes [21]. The review favored group-based education, finding significant improvements in HbA1c levels, body weight and systolic blood pressure (BP), fasting blood glucose (FBG), a decreased need for diabetes medication, and increased diabetes knowledge [21]. A subsequent publication in 2012, supported the findings of the former, favoring group-based education, with significant reductions in HbA1c, FBG, and body weight and improvements in diabetes knowledge compared with controls [22]. Similar findings were also noted in more recent systematic reviews [11, 23].

Several eminent studies [24, 25] have also attempted to develop and implement locally tailored and culturally sensitive DSME through peer-led approaches and found such approaches to be effective. A 2021 block randomized controlled trial showed significant improvements in self-management, self-efficacy, HbA1c, lipid profile, body weight, and BMI in older adults with diabetes. Peer-led self-management programs reduced healthcare worker workload and allowed older adults to learn self-management skills in the community [26]. In a study by Gallos et al. [27], a total of 207 Mexican Americans with HbA1c > 8% were randomized to receive either the Project Dulce peer intervention or continuation of standard diabetes care. The intervention group underwent eight weekly, 2-h diabetes self-management classes and subsequent monthly support groups, led by a trained peer educator. The intervention group exhibited significant improvements from baseline to month 10 in absolute levels of HbA1c (− 1.5%, *p* = 0.01), total cholesterol (− 7.2 mg/dL, *p* = 0.04), HDL cholesterol (+ 1.6 mg/dL, *p* = 0.01), and LDL cholesterol (− 8.1 mg/dL, *p* = 0.02). No significant changes were noted in the control group [27]. Similar results were noted in other trials [28–31].

Also, a recent meta-analysis evaluated the effectiveness, reach, uptake, and feasibility of digital health interventions for adults with type 2 diabetes, 26 studies (*n* = 4546 participants) in metanalysis. Overall, digital health intervention group participants had a − 0.30 (95% CI − 0.42 to − 0.19) percentage point greater reduction in HbA1c, compared with control group participants. The difference in HbA1c reduction between groups was statistically significant when interventions were delivered through smartphone applications (− 0.42% [− 0.63 to − 0.20]) and via SMS (− 0.37% [− 0.57 to − 0.17]), but not when delivered via websites (− 0.09% [− 0.64 to 0.46]) [32].

In view of the mounting quantum of evidence, peer-led approaches have gained popularity across the world. However, much of the evidence relates to isolated group-based sessions or peer-led approaches delivered through healthcare facilities or primarily digital interventions. None of the studies reviewed has combined group-based DSME delivered through a community leader with an analogous/complementary digital intervention. Also, there has been limited work from India towards improving the quality of diabetes care using a peer-led approach or digital interventions. The Kerala Diabetes Prevention Program did employ a low-cost-peer-led group education approach in favor of costlier ways. The study documented an improvement in cardiovascular risk factors and HRQOL [33]. Similarly, a trial by Kumar et al. [34] on 300 participants from a hospital in Mysuru found a LSM and medication reminder app to be effective in HbA1c reduction.

A positive result will set the template for a generalizable public health intervention with proven community effectiveness, sustainability, cost-effectiveness, and positive quality-of-life impact. While a negative result will require the testing of alternative approaches, it would still add substantially to existing knowledge on the subject. Given the diverse socio-cultural setting in which the trial is being proposed and the high power of the study, the results (positive or negative) should be widely applicable and have policy implications. The investigators will seek to pursue dissemination of the findings and if positive the refinement and expansion of the intervention to a wider variety of settings.

## Trial status

DEDINTT Protocol v 2.0 dated 01/03/2023.

Patient recruitment for the qualitative phase started on 6/7/23. Patient recruitment for phase 3 will be started on 1/2/24 and is expected to be completed 1/06/2025.

## Data Availability

The PI will have access to the final trial dataset. There are no contractual agreements that can limit data access and the investigators have unrestricted access. Any data required to support the protocol can be supplied on request. The corresponding author will make the datasets analyzed during the study, statistical code, and full protocol available on reasonable request.
